# Allogrooming, Self-Grooming, and Touching Behavior: Contamination Routes of Leaf-Cutting Ant Workers Using a Fat-Soluble Tracer Dye

**DOI:** 10.3390/insects8020059

**Published:** 2017-06-09

**Authors:** Roberto da Silva Camargo, Carolina Puccini, Luiz Carlos Forti, Carlos Alberto Oliveira de Matos

**Affiliations:** 1 Laboratório de Insetos Sociais-Praga, Departamento de Produção Vegetal, Faculdade de Ciências Agronômicas, UNESP, Caixa Postal 237, Botucatu, SP 18603-970, Brasil; carolpuccini.bio@gmail.com (C.P.); luizforti@fca.unesp.br (L.C.F.); 2 Campus Experimental de Itapeva, UNESP, Itapeva, SP18409-010, Brazil; carlos@itapeva.unesp.br

**Keywords:** *Atta sexdens*, leaf-cutting ants, allogrooming, self-grooming

## Abstract

The aim of this study was to determine whether worker self-grooming, allogrooming, and direct contact promotes the dispersal of substances among members of the colony. For this purpose, a tracer (Sudan III dye) was applied topically to a worker ant and the social interactions between the worker with the tracer and workers without the tracer were studied. Additionally, the worker heads were dissected to visualize whether or not the post-pharyngeal gland was stained. The post-pharyngeal glands from 50% to 70% of workers were stained depending on the size of the group. With the increase in the experimental group size, the frequency of interactions between workers increased, with touching being the most frequent behavior. The tracer dye was probably passed on by direct contact between workers, followed by self-grooming and allogrooming. These behaviors are responsible for the rapid dispersal of substances among colony members as observed in our experiment. The results therefore support the hypothesis that contact with substances promotes the contamination of nestmates, even in the absence of feeding, serving as a model for further studies on the contamination of workers with the active ingredients of insecticides.

## 1. Introduction

Although insecticides are commonly used to control leaf cutting ants, the distribution of baits between colony members is poorly understood. The contamination of ant workers can occur directly, i.e., during processing of the toxic bait for growth of the symbiotic fungus [1], although it is not exactly known how substances (e.g., insecticides) spread among members of a colony. An efficient way to disperse the active ingredient of an insecticide is the use of baits composed of citrus pulp, which is highly attractive to foragers.

Inside the nest, this bait is processed for growth of the fungus garden and it is postulated that workers are contaminated with the insecticide on that occasion [2]. After the baits become hydrated, they are deposited on the fungus garden surface and workers begin to cut in small pieces that are incorporated into the fungus garden. This process of incorporation starts 6 h after offering the baits and continues up to 18 h thereafter [2]. During this process, 70% of the workers become contaminated with the insecticide using a tracer dye [3]. The authors suggested that contaminated ants disperse sulfluramid insecticide through trophallaxis among adults as a chain reaction [2].Astrophallaxis is characterized by marked dilution, and it is important that the insecticide is potent enough to kill at low concentrations.

However, oral trophallaxis between adult leaf cutting ants is highly debated among myrmecologists: some authors claim that trophallaxis is rare [4], while some studies have shown oral trophallaxis in leaf-cutting ants [5].

The rate of trophallaxis varies widely among ant species, reflecting their phylogenetic position and feeding habits [6]. For example, in *Acromyrmex subterraneus subterraneus*, minima workers probably use oral trophallaxis to provide food to workers of the same size or larger [7]. However, in a more detailed study, the fluid intake rate of species of different feeding habits was quantitatively compared, and it was observed that workers of ant species that feed on the nectar and honeydew of homopterans, *Camponotus rufipes* (Formicinae) and *Pachycondy lavillosa* (Ponerinae), collected fluid at the highest intake rates, while workers of the leaf-cutting ant *Atta sexdens* (Myrmicinae) and a predator ant of the *Rhytidoponera impressa* (Ectatomminae) complex collected fluid at the lowest rate [8]. With respect to feeding habits, it was observed that species that collect fluids (nectar) during foraging, such as the genus *Camponotus* spp., possess a well-developed crop that is adapted to this foraging strategy. Once the forager has returned to the nest with the filled crop, it regurgitates and distributes the food through oral trophallaxis to nestmates [9]. On the other hand, a low fluid intake rate reflects other foraging strategies. In *Atta sexdens*, workers collect leaves for growth of the symbiotic fungus, ingest only extravasated fluid at the time of cutting and plant processing, and rarely visit nectar sources [10].

On the basis of this evidence, we suggest that trophallaxis occurs at low frequency or is absent in leaf-cutting ants [4]. It is known that trophallaxis is not the main trigger of the contamination of workers with insecticides in the colony and that intoxication occurs by direct contact with the toxic baits during their processing and incorporation into the fungus garden, as well as during allogrooming and self-grooming [11]. It can therefore be concluded that oral trophallaxis among adults is a controversial issue in leaf-cutting ants and an unlikely route for the exchange of substances between nest mates [5].

Given the above, the following question arises: How are workers contaminated? The first possibility is direct contact with the ant bait and its processing for growth of the symbiotic fungus. Another hypothesis is indirect contact with the active ingredient, by contact with contaminated individualsduring self-grooming, allogrooming, or touch. To test this hypothesis, an experiment involving different groups of workers was conducted. For this purpose, a tracer (Sudan III dye) was applied topically to a worker ant and the social interactions between the worker with the tracer and workers without the tracer were studied. Additionally, the heads of workers were dissected to visualize whether or not the post-pharyngeal gland was stained. The post-pharyngeal glands are involved in the production of digestive enzymes and specialized for lipid nutrition of adult leaf-cutting ants, i.e., absorbing, storing, metabolizing, and mobilizing lipids to the hemolymph. Consequently, the intensity of the color indicates the social interactions like self-/allogrooming or trophallaxis. A tracer dye was used to elucidate how substances are dispersed inside the nest.

## 2. Material and Methods

We hypothesized that indirect, i.e., individual–individual, contact promotes contamination through allogrooming or contact with contaminated individuals. To test this hypothesis, a tracer dye (Sudan III, Sigma-Aldrich, Steinhein, Germany) was applied topically to the pronotum of a worker at a concentration of 5% (w/w) in a soybean oil, and the social interactions between the contaminated worker and the non-contaminated group were studied in detail (4 treatments with 3 repetitions). The workers of *Atta sexdens* were transferred to a 250 mL plastic box containing a 1 cm layer of plaster at the bottom and a small amount (3 g) of the symbiotic fungus that belonged to the colony from which the workers were removed. A video camera (Sony) was positioned above the container for recording over a period of 24 h, and the videos were observed later. The study design consisted of 4 treatments with 3 repetitions, as follows:

(1) Group 1-1: 1 worker + 1 worker with tracer;
(2) Group 4-1: 4 workers + 1 worker with tracer;
(3) Group 9-1: 9 workers + 1 worker with tracer;
(4) Group 19-1: 19 workers + 1 worker with tracer.

The workers removed from the colonies, moving up with forceps, were selected according to size class based on a head length of 1.2–2.2 mm (medium size workers). Next, the pronotum of the individual was stained with the tracer dye, thus differentiated by color. The workers were marked with Edding^®^ marker pens in pink, white, and silver in their gaster. These pens were used because of their excellent adherence, rapid drying, and good visibility. This technique has been widely used for leaf-cutting ants [12]. After 24 h of recording, the workers were frozen (−10 °C) for subsequent dissection. For video analysis, we observed the following behavioral acts: direct contact—comprising physical interactions between the worker with the tracer and the other workers; self-grooming—comprising self-grooming of the worker with the tracer; allogrooming—comprising allogrooming of the worker with the tracer by other workers; touching—comprising touches between workers without the tracer; self-grooming 2—comprising self-grooming of workers without the tracer; allogrooming 2—comprising allogrooming between workers without the tracer. These behaviors were measured by frequency, i.e., the number of touches among workers.

**Figure 1:**
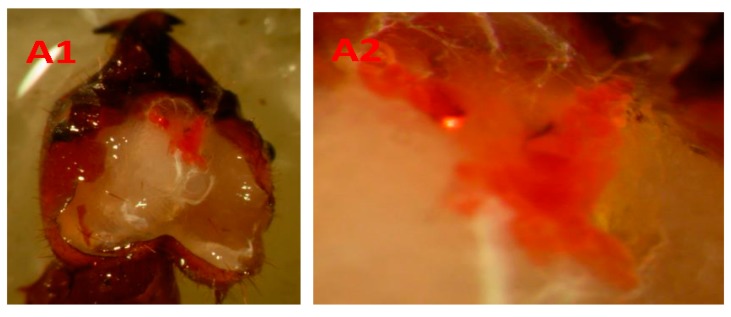

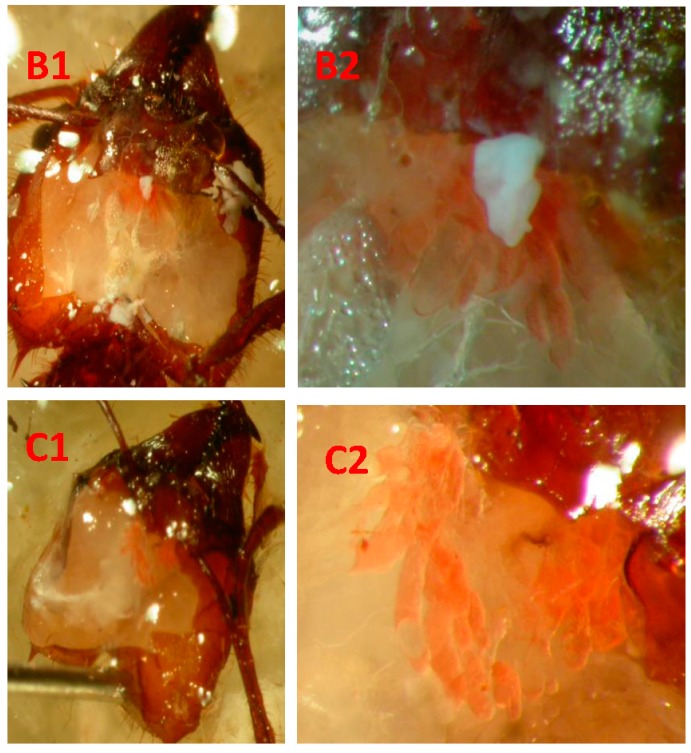
Dissection of *Atta sexdens* workers showing the exposed post-pharyngeal gland (60× magnification). (**A1**,**A2**) Worker ant that orally ingested 1 µL of a soybean oil solution containing 5% (w/w) Sudan III; (**A2**) Detail of the Sudan III-stained gland. (**B1**,**B2**) Worker ant in which 1 µL of a soybean oil solution containing 5% (w/w) Sudan III was applied to the pronotum; (**B2**) Detail of the Sudan III-stained gland; (**C1**,**C2**) Worker ant that remained for 24 h with the worker ant that received Sudan III on the pronotum at a proportion of 1:1 (5 with Sudan and 5 without Sudan); (**C2**) Detail of the gland stained with Sudan III in soybean oil solution at a concentration of 5% (w/w).

The heads of the workers were dissected with scissors and entomological forceps in physiological saline under a stereo microscope. The workers were categorized according to the presence or absence of a stained post-pharyngeal gland. The post-pharyngeal glands are involved in the production of digestive enzymes and specialized for lipid nutrition of adult leaf-cutting ants, i.e., absorbing, storing, metabolizing, and mobilizing lipids to the hemolymph. To guarantee the precision of dissection, preliminary experiments were carried out with ant workers in three situations (Figure 1) to determine whether the gland would be stained with a fat-soluble substance (Sudan III, Sigma-Aldrich).

The following variables were studied: (a) behavioral variables (frequency of self-grooming, allogrooming and worker–worker contact); (b) frequency of workers with stained post-pharyngeal gland. Behavioral analysis was performed by multiple comparison using ANOVA models. Each individual null hypothesis was specified by the linear combination of *p* parameters of the elementary model and *m* null hypotheses were tested simultaneously [13].

A logistic regression model of the frequency of stained and unstained workers was tested using Generalized Linear Model (GLM) with binomial variance and a logit link function. Additionally, logistic regression analysis of the frequency of stained individuals as a function of the proportion of individuals that received the dye was analyzed using GLM with Poisson variance and a logit link function. The same approach was used for logistic regression analysis of the frequency of unstained individuals as a function of the proportion of individuals that received the dye [14]. Statistical analyses were processed by R (Free Software Environment for Statistical Computing and Graphics) version 2.9.0 for Windows.

## 3. Results

Comparison of the groups within each behavior showed that group 19-1 differed significantly from groups 9-1 and 4-1 and, finally, group 1-1 (Table 1). When the behaviors were compared between groups, the behavioral acts touching 2 and self-grooming 2 were found to be more frequent and differed significantly in groups 19-1, 9-1 and 4-1 (Table 2). In contrast, in group 1-1, the behavioral acts self-grooming and touching were more frequent and differed from the other groups (Table 2).

A logistic regression model of the frequency of workers with stained and unstained glands was tested using GLM with binomial variance and a logit link function. The deviance (a measure of goodness-of-fit) of the model was non-significant (*p* > 0.05), indicating a lack of evidence against this model. No significant effects of the proportion of workers with unstained glands in relation to the proportion of workers with stained glands were observed for Groups 19-1 (*z* value = −0.654, Pr (>|*z*|) = 0.536), 9-1 (*z* value = −0.272, Pr (>|*z*|) = 0.760), or 4-1 (*z* value = 0.305, Pr (>|*z*|) = 0.760) (Table 3).

The logistic regression model of the frequency of stained individuals as a function of the proportion of individuals that received the dye was tested using GLM with Poisson variance and a logit link function. The deviance (a measure of goodness-of-fit) of the model (4.5212, d.f. = 8) was non-significant (*p* > 0.05). Significant effects (*p* < 0.05) of the proportion of stained individuals in relation to the proportion of individuals that received the dye were observed for Groups 19-1 (*z* value = 3.921, Pr (>|*z*|) = 8.82 × 10^−5^ (2.0794 times higher) and 9-1 (*z* value = 2.721, Pr (>|*z*|) = 0.00651) (1.5041 times higher). No significant effect was found for Group 4-1 (*z* value = 1.733, Pr (>|*z*|) = 0.083) (Table 4).

The same was observed for the logistic regression model of the frequency of unstained individuals as a function of the proportion of individuals that received the dye using GLM with Poisson variance and a logit link function. The deviance (a measure of goodness-of-fit) of the model (9.7118, d.f. = 8) was non-significant (*p* > 0.05), indicating a lack of evidence against this model. Significant effects (*p* < 0.05) of the proportion of unstained individuals in relation to the proportion of individuals that received the dye were observed for Groups 19-1 (*z* value = 3.606, Pr (>|*z*|) = 0.0003) (2.6391 times higher) and 9:1 (*z* value = 2.346, Pr (>|*z*|) = 0.0189) (1.7918 times higher). No significant effect was found for Group 4-1 (*z* value = 0.800, Pr (>|*z*|) = 0.4234) (Table 5).

In general, there were 4 workers with stained glands (66.7%) and 2 with unstained glands (33.3%) in Group 1-1, 11 workers with stained glands (73.4%) and 4 with unstained glands (26.6%) in Group 4-1, 18 workers with stained glands (60%) and 12 with unstained glands (40%) in Group 9-1, and 32 workers with stained glands (53.4%) and 28 with unstained glands (46.6%) in Group 19-1 (Figure 1).

## 4. Discussion

The present results corroborate the hypothesis that contact with insecticides (without feeding) can promote contamination in leaf-cutting ants, i.e., the behaviors of allogrooming and self-grooming as well as workers touching each other. Staining of the post-pharyngeal gland with the fat-soluble dye Sudan III simulates the ingestion of fat-soluble substances, including many insecticides [1,2]. The post-pharyngeal glands are involved in the production of digestive enzymes, although described as a gland of the salivary system, the post-pharyngeal gland displays characteristics of the foregut diverticulum [15,16]. This fact is supported by the presence of nematode parasites in its lumen [17,18].

Furthermore, it was showed that the secretion product of this gland is a determinant factor of colony odor [19]. However, it was demonstrated in enzymological and ultrastructural studies that the post-pharyngeal gland possesses characteristics of steroid hormone-producing cells that are rich in dividing mitochondria, peroxisomes, sites with cytochrome P450 (along the smooth reticulum of two types), numerous lipid droplets and derived mitochondria, which had transformed into fat deposits [20,21,22]. Recently, it was suggested that this organ (foregut diverticulum) is specialized for lipid nutrition of adult leaf-cutting ants, i.e., absorbing, storing, metabolizing, and mobilizing lipids to the hemolymph [23]. In view of this continuous deposition of lipids, the Sudan III dye was easily observed in the dissections of the present study (Figure 1).

With respect to the behavioral acts, a growing interaction was observed with an increasing number of individuals, with worker–worker contact being the most frequent behavior (Table 1). The tracer dye was probably dispersed by excessive contact between workers, followed by self-grooming and allogrooming (Table 2). Social insects are known for their conspicuous hygienic behavior demonstrated by many species to remove potentially pathogenic organisms present on their body surface and on nest mates, using self-grooming and allogrooming behaviors [6]. For example, allogrooming is effective in removing parasites such as *Metarhizium* from the cuticle and is directed at individuals exposed to parasites [24,25,26]. Curiously, however, ants tend to become immune to microorganisms such as *Metarhizium* as a result of exposure and contact by allogrooming [27]. On the other hand, self-grooming is a proactive behavior and is consequently stimulated by ants detecting the presence of individuals with microorganisms [28].

Another aspect related to these behaviors is their usefulness to discriminate between nest mates and non-nest mates. Nest mate recognition is based on a profile of specific compounds of the colony that is shared by all members, called the “gestalt” [29,30]. This colony gestalt is maintained by the continuous exchange of recognition signals through trophallaxis and allogrooming [31], and is affected by seasonal variation in the diet [32,33,34] and in the nesting substrate [35,36]. The grooming behavior seems to be fundamental for the passage of odor inside the colony [37] through the transfer of substances between workers, as observed in *A*. *subterraneus brunneus* [38].

As discussed above, self-grooming, allogrooming, and worker–worker contacthave different functions in the society of leaf-cutting ants, either hygiene and protection against microorganisms or the recognition of colony members. However, these behaviors are responsible for the rapid dispersal of substances between nest mates as observed in our experiment. We thus corroborate the hypothesis that indirect contact promotes the contamination of nestmates, serving as a model for further studies on the contamination of workers with the active ingredients of insecticides.

## 5. Conclusions

We conclude that self-grooming, allogrooming, and worker–worker contact were the main routes for dispersal of a fat-soluble substance to approximately half of the nest mates in our experiments, serving as a model for further studies on contamination of worker ants with insecticides containing fat-soluble active ingredients.

## Figures and Tables

**Table 1:** Frequency of behavioral acts (mean) executed by *Atta sexdens* workers.

**Self-Grooming**			**Self-Grooming 2**			**Worker–Worker Contact**		
19-1	70	a	19-1	791.33	a	19-1	428.66	a
9-1	64.33	a b	9-1	434	b	9-1	228.66	b
4-1	53	b	4-1	270.66	c	4-1	144.66	c
1-1	7.66	c	1-1	3	d	1-1	5.33	d
**Worker–Worker Contact**			**Allogrooming**			**Allogrooming 2**		
19-1	2693	a	19-1	25.66	a	19-1	138.66	a
9-1	698	b	9-1	21	a b	9-1	57.66	b
4-1	252.33	c	4-1	16.33	b	4-1	24	c
1-1	0	c	1-1	0.33	c	1-1	0.33	d

Values in the same column followed by different letters differ significantly from each other.

**Table 2:** Frequency of behavioral acts (mean) executed by *Atta sexdens* workers.

1-1			9-1			19-1			4-1		
Self-Grooming	7.67	A	Worker–Worker contact 2	698	A	Worker–Worker contact 2	2693	A	Self-Grooming 2	270.67	a
Worker–Worker contact	5.33	A b	Self-Grooming 2	434	B	Self-Grooming 2	791.33	B	Worker–Worker contact 2	252.33	a
Self-Grooming 2	3.0	B	Worker–Worker contact	228.67	C	Worker–Worker contact	428.67	C	Worker–Worker contact	144.67	b
Allogrooming	0.33	C	Self-Grooming	64.33	D	Allogrooming 2	138.67	D	Self-Grooming	53	c
Allogrooming 2	0.33	C	Allogrooming 2	57.67	D	Self-Grooming	70	E	Allogrooming 2	24	d
Worker–Worker contact2	0	C	Allogrooming	16.33	E	Allogrooming	25.67	F	Allogrooming	21	d

Values in the same column followed by different letters differ significantly from each other.

**Table 3:** Logistic regression model of the frequency of workers with stained glands/total workers.

Coefficients: Estimate Std. Error *z* Value Pr (>|*z*|)				
(Intercept)	0.6931	0.866	0.8	0.423
19:1	−0.5596	0.9039	−0.619	0.536
4:1	0.3185	1.0445	0.305	0.76
9:1	−0.2877	0.9428	−0.305	0.76

**Table 4:** Logistic regression model of the frequency of individuals with stained glands.

Coefficients: Estimate Std. Error *z* Value Pr (>|*z*|)				
(Intercept)	0.2877	0.5	0.575	0.56504
04:01	1.0116	0.5839	1.733	0.08317
09:01	1.5041	0.5528	2.721	0.00651
19:01	2.0794	0.5303	3.921	8.82 × 10^−5^

**Table 5:** Logistic regression model of the frequency of individuals with unstained glands.

Coefficients: Estimate Std. Error *z* Value Pr (>|*z*|)				
(Intercept)	−0.4055	0.7071	−0.573	0.56636
04:01	0.6931	0.866	0.8	0.42349
09:01	1.7918	0.7638	2.346	0.018978
19:01	2.6391	0.7319	3.606	0.000311
